# In silico and in vitro studies confirm Ondansetron as a novel acetylcholinesterase and butyrylcholinesterase inhibitor

**DOI:** 10.1038/s41598-022-27149-z

**Published:** 2023-01-12

**Authors:** Asma Gholami, Dariush Minai-Tehrani, Leif A. Eriksson

**Affiliations:** 1 Faculty of Life Sciences and Biotechnology, Shahid Beheshti University, Tehran, Iran; 2 Department of Chemistry and Molecular Biology, University of Gothenburg, 405 30 Göteborg, Sweden

**Keywords:** Medicinal chemistry, Theoretical chemistry, Drug discovery, Drug screening, Target validation, Biochemistry, Enzyme mechanisms, Neurochemistry

## Abstract

Alzheimer’s disease (AD) is a progressive neurodegenerative disorder that is growing rapidly among the elderly population around the world. Studies show that a lack of acetylcholine and butyrylcholine due to the overexpression of enzymes Acetylcholinesterase (AChE) and Butyrylcholinesterase (BChE) may lead to reduced communication between neuron cells. As a result, seeking novel inhibitors targeting these enzymes might be vital for the future treatment of AD. Ondansetron is used to prevent nausea and vomiting caused by chemotherapy or radiation treatments and is herein shown to be a potent inhibitor of cholinesterase. Comparison is made between Ondansetron and FDA-approved cholinesterase inhibitors Rivastigmine and Tacrine. Molecular docking demonstrates that interactions between the studied ligand and aromatic residues in the peripheral region of the active site are important in binding. Molecular dynamics simulations and binding pose metadynamics show that Ondansetron is highly potent against both enzymes and far better than Rivastigmine. Inhibitor activities evaluated by in vitro studies confirm that the drug inhibits AChE and BChE by non-competitive and mixed inhibition, respectively, with IC_50_ values 33 µM (AChE) and 2.5 µM (BChE). Based on the findings, we propose that Ondansetron may have therapeutic applications in inhibiting cholinesterase, especially for BChE.

## Introduction

Alzheimer’s disease (AD) is one of the most prevalent and irreversible neurodegenerative disorders affecting elderly, and is characterized by different types of gradual symptoms, from fluctuations in behavioral and social skills to memory loss. Eventually, severe aspects of disability to perform daily routine activities will appear in the patient^1^. More than 20 million individuals worldwide suffer from this dementia, a number that is expected to increase with the growth of the elderly population in the future^2,3^.

In spite of the fact that there is no clear understanding of AD pathogenesis, both genetic and environmental factors play an important role in AD development^4^. Multiple hypotheses, such as the amyloid-beta oligomer hypothesis, the cholinergic hypothesis, and the tau hypothesis have been presented, aiming to shed light on AD progression and to identify new therapeutic approaches against AD^5^.

According to the cholinergic hypothesis, the levels of Acetylcholine (ACh) and Butyrylcholine (BCh) which have neurotransmitter functions, are decreased in the brain regions of AD patients^6^. The absence of these neurotransmitters disrupt the conduction of electrical impulses through the nerve cells by reducing the cholinergic signaling and neurotransmission in the brain. As a result, severe cell damage and memory loss will occur due to the improper function of the brain^2,7^.

**Figure 1:**
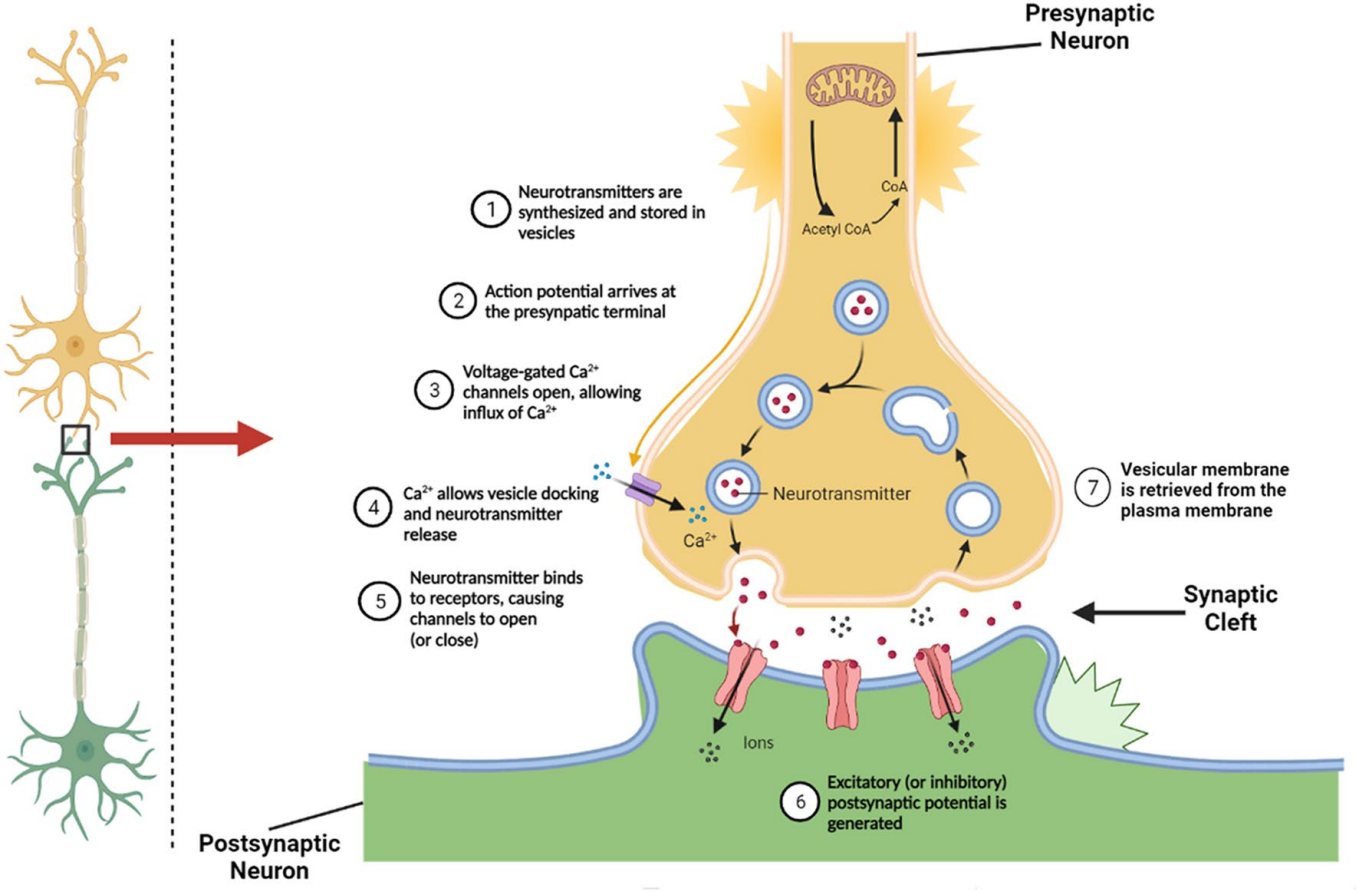
Synaptic pathways and role of Acetylcholine and Cholinesterase in a normal nerve cell (the figure was created with BioRender.com).

As seen in Fig. 1, the neurotransmitters are packed into vesicles and released into the synaptic cleft. The receptors accept the neurotransmitters in the postsynaptic neuron and rapidly cleave them into choline and acetate by the two enzymes Acetylcholinesterase (AChE) and Butyrylcholinesterase (BChE)^8^, widely distributed in the central nervous system^4,9^. Choline will subsequently be recycled into a new neurotransmitter for the next message^8^.

It has been observed that in the brains of patients with AD, overexpression of these enzymes and lack of ACh/BCh occurs and leads to reduced communication between neuron cells. Consequently, approaches that inhibit AChE and/or BChE may hinder AD progression^6^.

Many attempts have been made to discover more effective drugs for reducing the symptoms and slowing down the development of AD. For cholinesterase, the main FDA-approved inhibitors are Donepezil, Galantamine, Huperzine, Rivastigmine, and Tacrine^6,10^, summarized in Table1^11,12^.

**Table 1:** Characteristics and properties of approved cholinesterase inhibitors.

Compound and PubChem CID	Mechanism of inhibition	Penetration through blood–brain barrier	2D structure
Rivastigmine 77991	Non-competitive	Good	
Tacrine 1935	Non-competitive	Good	
Galantamine 9651	Competitive	Good	
Donepezil 3152	Non-competitive	Good	
Huperzine 1253	Non-competitive	Good	

The mechanism of action of Tacrine and Rivastigmine is to block the catalytic site of cholinesterases; of these, Rivastigmine furthermore induces a structural change in the binding pocket of the enzymes. Both are inhibitors of AChE as well as BChE and easily cross the blood–brain barrier^6,13^. Donepezil blocks the catalytic site of AChE by forming a hydrogen bond and an aromatic interaction in the active site but does not inhibit BChE^13^.

Galantamine is an alkaloid compound isolated from the bulbs and flowers of *Galanthus woronowii*, belonging to the Amaryllidaceae family. It has lower toxicity and potency, and releases ACh by allosteric modulation of nicotinic acetylcholine receptors and inhibition of AChE. Huperzine is another alkaloid derived from the Chinese herb *Huperzia serrata*, and is a potent, reversible, selective inhibitor of AChE^2,13^.

The main issue in stopping the development of AD is that the efficacy and performance of these agents are still not perfect^13^. Moreover, significant adverse effects, such as gastrointestinal disturbance, hepatotoxicity, syncope, sleep disturbances, and hypotension have also been documented. Therefore, searching for more potent and stronger agents with less adverse effects is highly relevant for improved treatment of this disease^12^.

**Figure 2:**
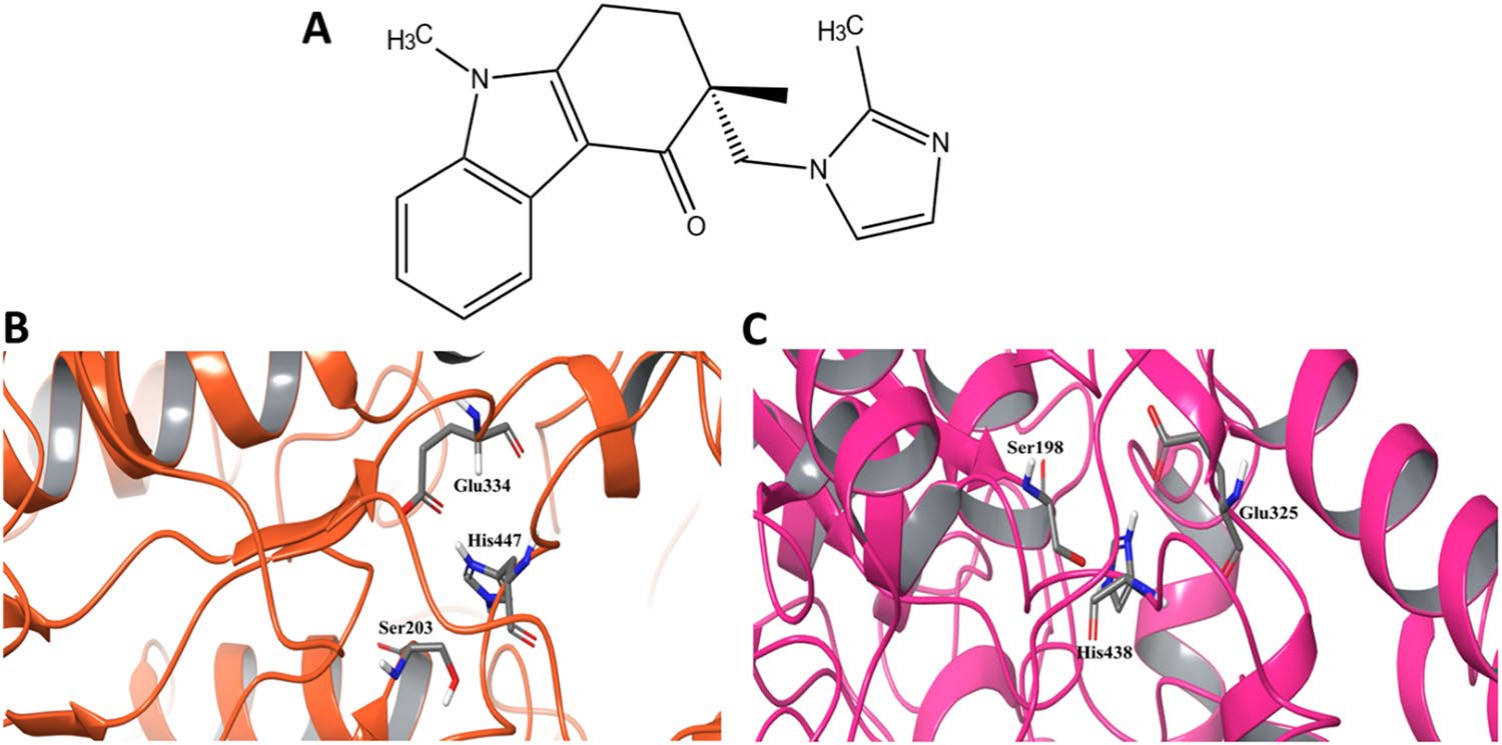
(**A**) Chemical structure of Ondansetron. (**B**,**C**) Active sites of (**B**) AChE, and (**C**) BChE, with key active site residues indicated.

Ondansetron (trade name Zofran) is a type 3 serotonin (5-hydroxytryptamine) receptor (5-HT3) antagonist (Fig. 2A), which is prescribed against vomiting and nausea caused by chemotherapy or radiation treatment^14,15^. In comparison with other antiemetic drugs, the side effects are moderate, with high safety and efficacy. It is also used as an antiemetic drug for the first trimester of pregnancy, as well as in relief of opioid- and alcohol-induced pruritus, anxiety disorders, withdrawal syndrome, and gastrointestinal motility disorders^16,17^.

Some studies have explored the effect of Ondansetron on cholinesterase inhibition. For example, the combination of Ondansetron with Pyridostigmine as an organophosphorus pretreatment compound showed significant decrease in AChE activity in red blood cells of guinea pigs^18^.

It has also been demonstrated that the 5-HT3 receptor antagonist Ondansetron together with the FDA approved drugs Donepezil potentiates the effects of AChE inhibition on the neuronal network oscillations in the rat dorsal hippocampus^19^.

Another usage of Ondansetron as a therapeutic agent is in the treatment of psychiatric disorders which involve abnormalities of interception and associated neural circuitry centered on the insula. Different doses of Ondansetron were applied for patients whereby it was finally suggested that 24 mg of this drug modulates the hyperactivity in these regions^20^.

Regarding BChE inhibition, recent in silico and in vitro studies of a new series of Tacrine derivatives (spiro[chromeno[4,3-b]thieno[3,2-e]pyridine]-7-amines) showed that these exerted good BChE inhibitory activity, thereby proving to be promising candidates for evaluation in synthetic AD models^21^.

Based on the above findings, it is clear that Ondansetron can affect the nerve system. As a result, this study aims to explore Ondansetron as an inhibitor of cholinesterases. As a first step, different in silico approaches were applied to investigate the interaction of Ondansetron with AChE and BChE. Comparison was made between this drug in both systems with the two FDA-approved cholinesterase inhibitors Rivastigmine and Tacrine (Table 1) to evaluate its efficacy. In vitro and kinetics studies of the inhibition strongly confirm the computational model. The calculation of K_i_ and IC_50_ values for these enzymes along with Arrhenius plots verify the drug binding and modes of action, and indicates that Ondansetron has stronger affinity towards BChE compared to AChE. Moreover, our study shows that Ondansetron is more potent than Rivastigmine in both enzymes but less so compared to Tacrine. For the latter, however, severe adverse effects including hepatotoxicity has led to its withdrawal in many countries^11^; thus new and safer drugs targeting cholinesterases is of significant importance. To validate the desired properties of Ondansetron, in vivo studies must be performed to confirm its strength, reliability, longevity, efficiency, and potency.

## Materials and methods

### In silico studies

#### Protein preparation

The three-dimensional crystal structures of the target enzymes AChE (PDB ID:4M0E)^22^ and BChE (PDB ID:5DYW)^23^ were downloaded from the Protein Data Bank (PDB) (http://www.rcsb.org). The proteins were prepared and refined using the Protein Preparation Wizard in Maestro (Schrödinger 2021-4, http://www.schrodinger.com). Bond orders were assigned during the preprocessing stage of the crystal structures, and after retrieving missing loops or side chains, all water molecules beyond 3.0 Å were deleted from the system. Protein hydrogen bond assignments were optimized and protonation states at pH 7 determined using PROPKA^24^. Finally, a restrained minimization with the OPLS4 force field^25^ was performed with an RMSD convergence of heavy atoms of 3.0 Å.

#### Ligand preparation and docking

All ligand structures were downloaded from the PubChem database (http://www.pubchem.ncbi.nlm.nih.gov) and transferred into Maestro Schrödinger using LigPrep. The Epik module^26^ was used for possible ionization states at physiological pH 7.0 ± 2.0, and the OPLS4 force field was selected for the optimization. In order to perform the molecular docking and predict the interactions of the protein–ligand complexes, the Schrödinger Induced Fit Docking (IFD) methodology was used^27,28^. All three inhibitors were docked towards the active sites of both AChE and BChE. The grid box for the docking was defined at the centroid of the binding site. During the initial docking procedure, the van der Waals scaling factor was set at 0.5 for both receptor and ligand. The Prime refinement step was set on side chains of residues within 5 Å of the ligand. No constraints were applied, and all remaining parameters were set to default.

#### PASS prediction

The PASS (Prediction of Activity Spectra for Substances) predictions of Ondansetron, Rivastigmine and Tacrine were carried out using the PASS-Way2Drug server (http://www.pharmaexpert.ru/passonline/), using canonical SMILES from the PubChem server (https://pubchem.ncbi.nlm.nih.gov/)^29^. In the PASS predictions, the probability to be active (Pa) was kept greater than 0.3, and compared with the probability to be inactive (Pi). Four possible biological activities were predicted.

#### Molecular dynamics (MD) simulations

The stabilities of the ligand–protein complexes were investigated through MD simulations for 200 ns, using the Desmond engine^30^ in Schrödinger (Schrödinger 2021-4, http://www.schrodinger.com). The TIP3P force field was used to model water molecules^31^. Periodic boundary conditions were applied with a 10 Å water buffer around the protein in a cubic simulation box. Na^+^ and Cl^−^ ions were added to neutralize the system and to give a final NaCl concentration of 150 mM. The OPLS4 force field was used for the proteins and ligands. The isothermal–isobaric (NPT) ensemble was used, and for adjusting the temperature and pressure of the systems, the Nose Hoover thermostat^32^ and the Martyna–Tobias–Klein barostat^33^ were employed at 300 K and 1.01325 bar, respectively. All data analyses such as calculation of root mean square deviations (RMSD), root mean square fluctuation (RMSF), and protein–ligand contacts were obtained from the simulation interaction diagram (SID) program in Schrödinger 2021-4 (http://www.schrodinger.com).

#### Clustering

To obtain representative structures for the Binding Pose Metadynamics simulations (BPMD), Desmond Trajectory clustering in Maestro (Schrödinger 2021-4) was used, based on the obtained MD trajectories. The lowest energy structures from the most populated clusters were selected for the BPMD calculations.

#### Binding pose metadynamics (BPMD)

Binding pose metadynamics was performed using a set biasing force, to explore how stable ligands are in the binding pocket of the receptor. Weaker ligands will experience higher fluctuations with larger RMSD values in comparison with the more stably bound ones^34^.

BPMD was implemented using the Desmond engine^30^ in Schrödinger 2021-4 (http://www.schrodinger.com), with the OPLS4 force field. Before the actual metadynamics run, the system was solvated in a box of TIP3P water molecules followed by several minimization and restrained MD steps to allow the system to slowly reach the desired temperature of 300 K, as well as releasing any bad contacts and/or strain in the initial starting structure^33^. 10 independent BPMD simulations of 10 ns were performed for each system, using the Root-Mean-Square Deviation (RMSD) of the ligand heavy atoms relative to their starting position as the Collective Variable (CV).

### Experimental studies

#### Sample collection

A blood sample was obtained from a healthy volunteer. The protocol was approved by the Human Ethics Committee of Shahid Beheshti University (SBU) of Iran with the approval number of IR.SBU.REC.1401.050. All research was performed in accordance with relevant guidelines/regulations, and informed consent was obtained from all participants. The volunteer had no significant medical disorder, was not taking any previous medication for at least 30 days, had no history of alcohol, drug or cigarette abuse, and no recurrent or a past history of psychiatric illness. From the volunteer, 5 ml of blood was collected in vacutainer tubes.

#### Acetylcholinesterase preparation

AChE of the erythrocytes is bound to the plasma membrane. The blood sample was treated with EDTA (1.5 mg/ml) as anticoagulant agent and centrifuged at 3000*g* for 5 min to precipitate the erythrocytes. The plasma and buffy coats were removed and discarded. Erythrocytes were washed twice with isotonic solution (NaCl 0.15 M), each time centrifuged to precipitate the intact cells. Erythrocytes were lysed by adding a hypotonic solution (NaCl 0.01 M). The solution was centrifuged at 15,000*g* for 20 min and the supernatant, which contained hemoglobin and other cell contents, was removed. The precipitate was washed with phosphate buffer 0.1 M, pH 7, and centrifuged at 15,000*g* for 20 min. The final precipitate (cell membrane) was collected for the AChE assay.

#### Butyrylcholinesterase preparation

BChE is present in the blood serum. The blood sample was allowed to clot without adding any anticoagulants, and then centrifuged at 3000*g* for 5 min. The serum as a source of BChE was separated and stored at − 20 °C for further use.

#### Enzyme assay

Both AChE and BChE were assayed according to the Ellman colorimetric method^35^. In brief, the assay tube contained 1500 µl 0.1 M phosphate buffer at pH 7, 50 µl 50 mM acetylthiocholine, 50 µl 10 mM DTNB (5,5′-dithiobis-2-nitrobenzoic acid), and 20 µl of either blood serum as a source of BChE, or plasma membrane solution as a source of AChE. The assay was performed either in the absence or presence of Ondansetron in the concentration range 8–84 µM. The yellow reaction product TNB (5-thio-2-nitrobenzoic acid) was monitored at 410 nm using a UV–Visible 1240 Shimadzu spectrophotometer and the activities of the enzymes calculated using the TNB extinction coefficient 13,600 M^−1^ cm^−1^.

The effect of pH and different temperatures on the enzyme activity was also measured in the absence or presence of the drug with the final concentration of 21 µM. Lineweaver–Burk plots were used to determine the kinetic parameters and inhibition types. Arrhenius plots were used to compare the activation energies of the enzymes in the presence or absence of the drug by calculating Vmax of the enzymes at different temperatures^36,37^.

The protein content of the samples was measured using the Lowry method^38^, with casein used for the standard curves.

## Results and discussion

### In silico studies

#### Molecular docking

The structure of AChE contains several important parts such as the esteratic site which harbors the catalytic triad Ser203, His447, and Glu334 (Fig. 2B), located in a 20 Å deep narrow gorge containing several conserved amino acids. A second part is the peripheral anionic site (PAS) which extends beyond Tyr337 at the catalytic/peripheral site interface to the entrance of the gorge, and contains several aromatic side chains (e.g., Tyr72, Trp86, Tyr124, Phe295, Tyr337 and Phe338). Kinetic and thermodynamic studies have shown that inhibitors can interact with either or both of the two binding regions^22^. In BChE, the catalytic triad located at the bottom of the 20 Å gorge is made up by Ser198, His438, and Glu325 (Fig. 2C)^39^. Four aromatic residues in the peripheral site, Trp82, Trp231, Tyr 332 and Phe329, plus Asp70, have been found to be important for ligand binding^40,41^.

Alignment was made between the amino acid sequences (Fig. S1), aiming to compare the active site residues in both enzymes using BChE as reference. The sequence identity and similarity between the two proteins are 55% and 70%, respectively, and the identity of the active site residues (Ser-Glu-His) is conserved (Fig. S1). Knowledge of the structures and conserved residues of the two enzymes is essential for compound selection and determination of binding modes of the cholinesterase inhibitors.

To validate the robustness of Glide used for the docking, re-docking of the co-crystallized ligands was performed. The crystal structure of AChE (PDB ID: 4M0E^22^) with Dihydrotanshinone I as ligand and BChE (PDB ID: 5DYW^23^) with the ligand N-{[(3S)-1-benzylpiperidin-3-yl]methyl}-N-(2-methoxyethyl)naphthalene-2-sulfonamide were selected for redocking. Analyzing the root mean square deviation (RMSD) results for the compounds in AChE and BChE, respectively, gave the values 1.276 Å and 1.570 Å for the two ligands, thus confirming the validity of the methodology used. Moreover, the docked poses of both ligands overlay perfectly with their crystal structure conformations (Fig. S2).

The active sites of holoenzymes of AChE and BChE were defined based on the co-crystallized ligands (Fig. 2B,C)^40^ followed by Induced Fit Docking (IFD) of the ligand set containing Ondansetron and the two FDA approved cholinesterase inhibitors Tacrine and Rivastigmine (Table 1). Table 2 shows the docking results and free energies of binding calculated using MMGBSA for both enzymes. The trends between the docking scores and MMGBSA energies are highly consistent, and indicate that Tacrine is the strongest binder in both systems, and Rivastigmine the weakest. Ondansetron seems to bind better to BChE, whereas Rivastigmine is more potent towards AChE.

**Table 2:** Data from docking analyses and free energies of binding of different ligands for both receptor proteins.

	Docking score (kcal/mol)	MMGBSA (kcal/mol)
**AChE**		
Ondansetron	− 6.364	− 50.88
Tacrine	− 6.563	− 58.15
Rivastigmine	− 5.616	− 47.43
**BChE**		
Ondansetron	− 6.954	− 54.14
Tacrine	− 7.171	− 58.44
Rivastigmine	− 5.282	− 22.96

**Figure 3:**
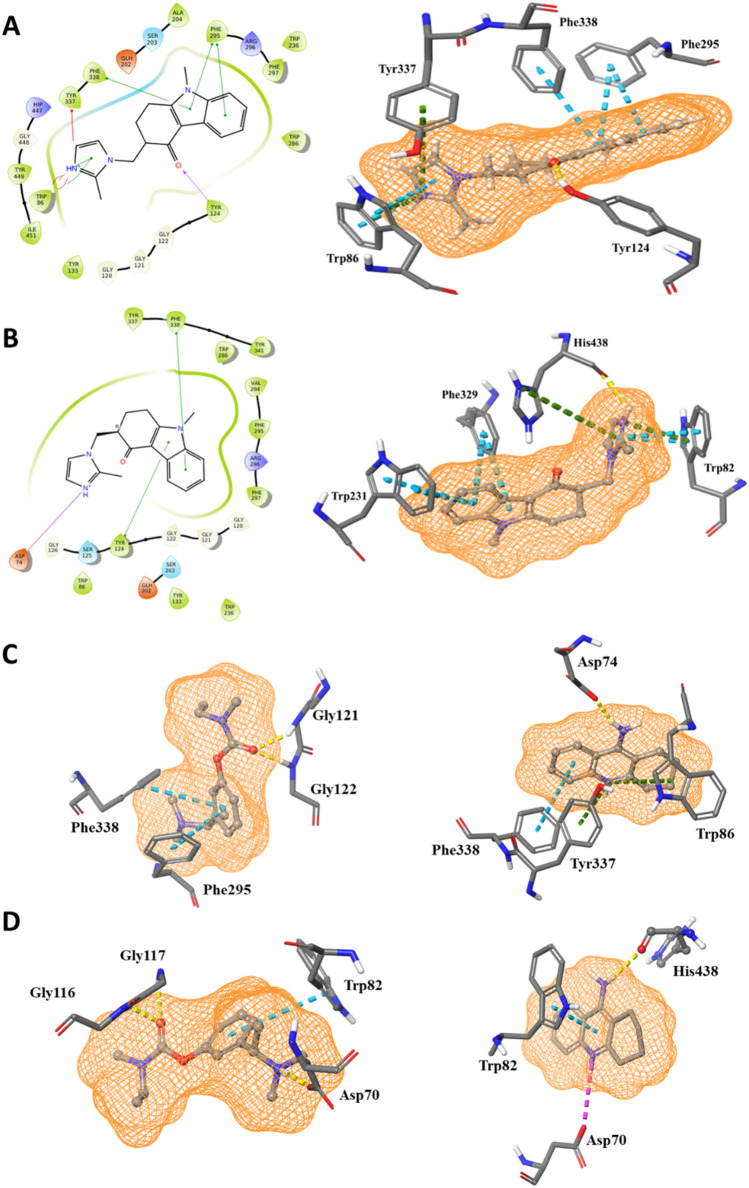
2D interaction diagrams (left) and atomic contacts (right) of Ondansetron docked to (**A**) AChE and (**B**) BChE. Atomic contacts of Rivastigmine (left) and Tacrine (right) docked to (**C**) AChE and (**D**) BChE. Different types of interactions are represented by color-dashed lines: blue, yellow, purple and green denote π–π stacking, hydrogen bonds, ionic bond, and π cation bonds, respectively.

Figure 3A shows the interactions of Ondansetron docked in the active site of AChE. Ondansetron forms π–π stacking interactions with the aromatic rings of Phe295, Phe338, and Trp86. In addition, Trp86 displays one π-cation interaction. Tyr124 and Tyr337 forms hydrogen bonding and π-cation interactions, respectively. There are no direct interactions between the ligand and residues of the catalytic site; however, as outlined above, some aromatic residues in the peripheral site located at the entry to the active gorge are responsible for binding many inhibitors and also contribute to the catalytic efficiency and activity of the enzymes. Among these, Trp86 and Tyr337 are the two major aromatic residues that are involved in binding to the ligands^22,40^. Our docking results suggest a non-competitive inhibition for Ondansetron through allosteric site binding, resulting in reduced efficacy of the enzyme.

Non-competitive binding modes were also observed for Tacrine and Rivastigmine (Fig. 3C). Tacrine forms a hydrogen bond with Asp74, π–π stacking interaction with Phe338, and two π cation bonds with Trp86 and Tyr337. In addition, π–π stacking interactions with Phe338 and Phe295 were observed for Rivastigmine, as well as two hydrogen bonds with Gly121 and Gly122.

In BChE (Fig. 3B), one hydrogen bond and one π-cation interaction are predicted to be formed between Ondansetron and one of the main catalytic residues, His438. The most significant residues to have interactions with ligands in the peripheral site of BChE are Trp82, Trp231, and Phe329^41^. This is in accord with the current study, where we see that Trp82 forms π-cation interaction with Ondansetron, and this amino acid plus Trp231 and Phe329 form π–π stacking interactions with the aromatic rings of the inhibitor. The interaction with His438 in the binding pocket and the residues at the peripheral site would suggest that Ondansetron binds to BChE through mixed inhibition, i.e., binding the active site of the enzyme (competitive inhibition) and the enzyme–substrate complex (noncompetitive inhibition) with different affinity. In comparison, Rivastigmine forms one π–π stacking interaction with Trp82, and three hydrogen bonds with Asp70, Gly116 and Gly117. For Tacrine, there is π–π stacking interaction with Trp82, an ionic bond with Asp70, and a hydrogen bond with the main catalytic residue of His438 (Fig. 3D).

From the free energy of binding values for Ondansetron and the two FDA-approved drugs in AChE and BChE (Table 2), we note that Ondansetron and Tacrine bind most strongly to the holo form of the enzyme. However, to gain better insight on the possible activities of the compounds and the stabilities of the formed complexes, PASS analysis, MD and BPMD simulations were conducted as follows.

#### PASS prediction

The prediction of activity spectra for substances (PASS prediction) was conducted for Ondansetron with respect to four relevant biological activities. The PASS prediction results of the selected ligand along with the positive controls Tacrine and Rivastigmine are listed in Table 3.

**Table 3:** PASS predictions of biological activities^a^ for Ondansetron Tacrine and Rivastigmine.

Biological activities	Ondansetron		Tacrine		Rivastigmine	
	Pa	Pi	Pa	Pi	Pa	Pi
1. Acetylcholinesterase inhibitor	–	–	0.267	0.004	0.020	0.012
2. Butyrylcholinesterase inhibitor	0.062	0.036	0.376	0.003	0.062	0.072
3. Alzheimer's disease treatment	0.423	0.024	0.341	0.047	0.283	0.073
4. Neurodegenerative diseases treatment	0.290	0.145	0.442	0.088	0.483	0.068

^a^Pa: Predicted probability of substance being active. Pi: Predicted probability of substance being inactive.

There are three activity levels indicated by the PASS program. If Pa > 0.7, the substance is very likely to exhibit experimental activity; if 0.5 < Pa < 0.7, the substance is likely to exhibit the activity but with less probability; If Pa < 0.5, the substance is unlikely to exhibit. However, if activity is confirmed experimentally despite low Pa value, the substance may be a ‘new chemical entity’ not catered for in the training of the PASS algorithm^43,44^. As can be seen from Table 3, none of the compounds is predicted to be active, or even moderately active, despite two of them being clinically proven and approved for the treatment of AD. No activity was predicted by this program for Ondansetron as Acetylcholinesterase inhibitor and a very low value was found for Butyrylcholinesterase—similar to that of Rivastigmine. However, given the experimental evidence (see below), Ondansetron can in this context be labeled a “new chemical entity”. A closer look at the table indicates that Ondansetron displays higher active possibility for the treatment of AD in comparison with Tacrine and Rivastigmine. For neurodegenerative disease treatment the PASS data for Tacrine and Rivastigmine are similar (∼ 0.4), and slightly higher than the value for Ondansetron (∼ 0.3). Taken together, it hence seems plausible that Ondansetron is a good candidate for dementia treatment.

#### Molecular dynamics simulations

The stability and accuracy of the different protein–ligand complexes obtained from the docking studies were explored through 200 ns MD simulations.

**Figure 4:**
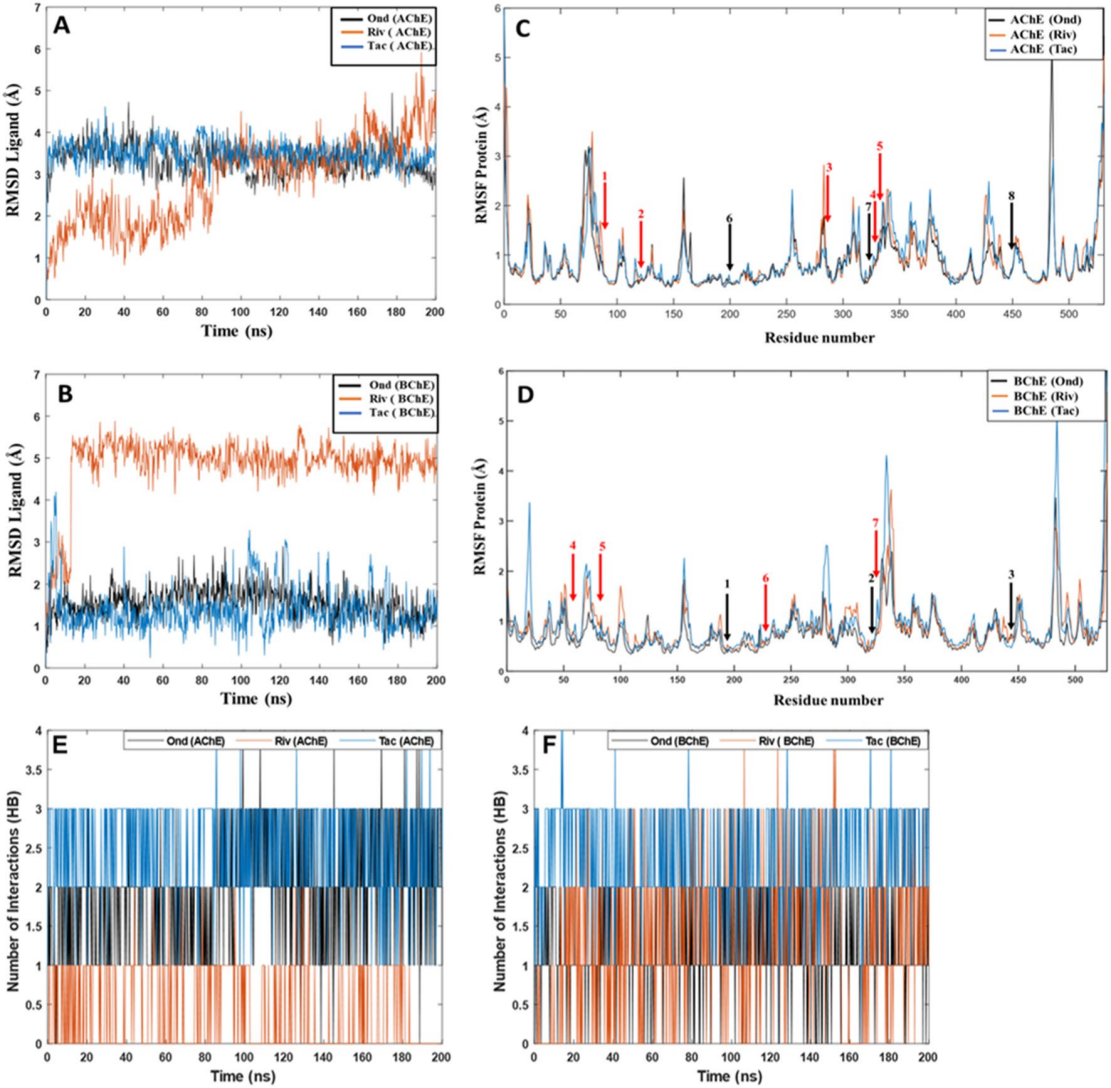
Root-mean-square deviation (RMSD) values of the AChE and BChE ligand complexes during 200 ns MD simulations: (**A**) Ondansetron, Tacrine, and Rivastigmine as ligands in AChE; (**B**) Ondansetron, Tacrine, and Rivastigmine as ligands in BChE. Root mean square fluctuation (RMSF) values of the AChE and BChE proteins during 200 ns MD simulations; (**C**) AChE containing Ondansetron, Rivastigmine, and Tacrine as ligands; (**D**) BChE containing Ondansetron, Rivastigmine, and Tacrine as ligands. Red and black arrows with numbers correspond to the location of peripheral residues and catalytic residues, respectively. (**E**) Hydrogen bonds between Ondansetron, Tacrine, and Rivastigmine as ligands in AChE; (**F**) Ondansetron, Tacrine, and Rivastigmine as ligands in BChE.

The RMSD data for the ligands binding to AChE (Fig. 4A) shows a continuous increase throughout the simulation for Rivastigmine, indicating that this ligand may be more prone to dissociate. We find that, despite the ligand remaining largely in the binding cavity of the protein and still has interactions with some crucial amino acids such as Trp86 in the last snapshot of the MD simulation, the changes are significant with part of the aromatic ring and the alkylamine substituent (Table 1) exposed to the solvent and undergo large fluctuations. This is in agreement with the lower docking score and MMGBSA values (Table 2) for Rivastigmine. Our results suggest that there are no considerable changes in RMSD values for Ondansetron or Tacrine, and that both form stable complexes with AChE.

The RMSD graphs for BChE (Fig. 4B) show that all three inhibitors have preserved their binding affinity and are still tightly coupled to their respective binding sites. Ondansetron lies below ∼ 2 Å and no significant fluctuations were observed throughout the simulation period, implying that the binding of the ligand to the active site of BChE is very stable and strong. As in AChE, Tacrine in BChE displays slightly lower RMSD values compared to our target ligand; however, analysis of the MD trajectory shows that part of the ligand is exposed to the solvent which accounts for the minor fluctuations seen in the graph during the simulation. Analysis of the MD trajectory for Rivastigmine shows that the ligand remained stable in the binding pocket after an initial large movement/adjustment as noted by the large jump in RMSD at t = 10 ns. The MMGBSA binding energy calculations (Table 2), show higher binding affinities for Ondansetron than Rivastigmine in BChE.

We also analyzed the protein root-mean-square-fluctuations (RMSF), showing which residues that fluctuate the most during the MD simulation (Fig. 4C,D). The RMSF values of AChE with any of the ligands bound remain relatively stable (Fig. 4C). The highest variation is related to residues 482–486, when the protein had Ondansetron as ligand. These are part of a loop close to the C-terminal and are not involved with the active site of the protein. The red arrows in the graph indicate the location of the key amino acids in the peripheral site (1, 2, 3, 4, 5 for Trp86, Tyr124, Phe295, Tyr337 and Phe338, respectively). For AChE with Rivastigmine bound, some fluctuations are observed for residues 281–284, and very close to Phe295 (arrow number 3), indicating that the interaction of the ligand with these parts creates changes to the protein. Except for these parts, the protein was highly stable throughout the simulations, irrespective of ligand. We also note that the catalytic residues (black arrows 6, 7, and 8) display very low RMSF values during all the simulations.

The RMSF values for BChE with Ondansetron bound (Fig. 4D) indicate that this complex is quite stable during the whole simulation time, while for Rivastigmine and Tacrine some fluctuations are observed. There are two regions with large peaks close to amino acids in the binding site: residues 70–77 corresponding to a loop including Asp70 in peripheral site (arrow 4) and Trp82 (arrow 5); and residues 334–341 corresponding to a loop very close to Phe329 (arrow 7). For the BChE–Rivastigmine complex, residues 101–105 show large fluctuations; this is a region that is far from the binding pocket. For the system with Tacrine bound, two large peaks are seen near the C- and N-termini, far from the active site; as is also the region with residues 281–285. The location of the three main residues in the catalytic pocket, Ser198, Glu325 and His438, respectively, are shown by black arrows. These residues as well as Trp231 (arrow 6) were found to remain highly stable during the MD simulations of BChE.

Further examination of the system stability was conducted by probing the evolution of interactions between ligand and protein, here measured by the formation of hydrogen bonds during the MD simulations. Ondansetron bound to AChE maintained an average of 1.90 hydrogen bonds, whereas Rivastigmine and Tacrine had an average of 0.45 and 2.47 hydrogen bonds, respectively (Fig. 4E). This indicates that our targeted ligand is more stably bound than Rivastigmine, in agreement with the RMSD plots in Fig. 4A.

In the case of ligands bound to BChE, the average number of hydrogen bonds for Ondansetron is 1.22 while for Rivastigmine and Tacrine it is 1.28 and 2.38, respectively (Fig. 4F). This confirms that Tacrine interacts stronger than the other ligands in this system; for Ondansetron and Rivastigmine the stability and number of hydrogen bonds are very similar.

#### Binding pose metadynamics (BPMD)

Next, we performed binding pose metadynamics (BPMD) simulations for all six systems. The pose stability was evaluated based on the PoseScore, i.e., the RMSD of the ligand with respect to the initial ligand heavy atoms coordinates. The threshold value for ligand stability in the binding pocket is a PoseScore ≤ 2 Å^34^.

**Figure 5:**
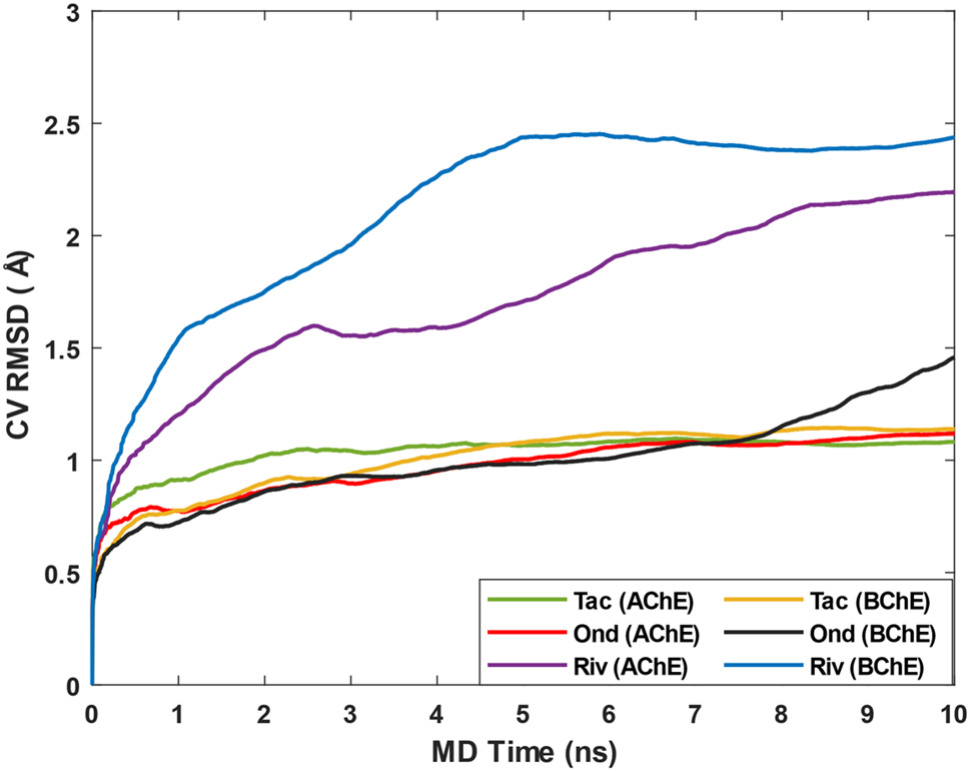
RMSD estimates averaged over 10 BPMD runs vs simulation time.

Figure 5 shows that when Ondansetron is located in AChE the calculated values of PoseScore is 1.10 Å, indicative of stable binding. For Tacrine in AChE, the obtained PoseScore is 1.03 Å, and for Rivastigmine 2.15 Å with a large increase in value, indicating lower ligand stability in comparison with the other two ligands. When Ondansetron is bound to BChE, the PoseScore value is 1.29 Å and for Tacrine and Rivastigmine it is 1.13 Å and 2.39 Å, respectively. Based on these results and the RMSD data (Fig. 4A,B) and the energetics reported in Table 2, we conclude that Ondansetron can be a more potent candidate against cholinesterase compared to Rivastigmine; however, less so than Tacrine in both proteins.

### Experimental studies

#### Binding of Ondansetron to the enzymes

**Figure 6:**
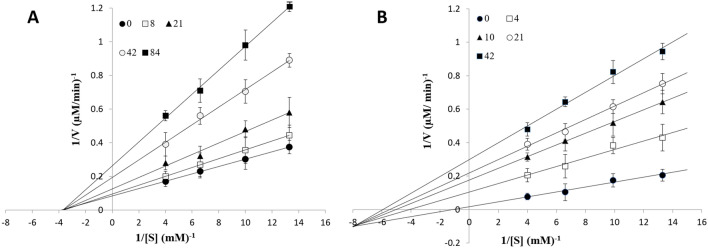
Double reciprocal plot determining the type of inhibition by Ondansetron in (**A**) AChE (non-competitive inhibition), and (**B**) BChE (mixed inhibition).

The effect of Ondansetron on AChE and BChE activity was investigated through different in vitro assays, and the type of inhibition and the kinetic factors of the binding calculated. The enzymatic activities of AChE and BChE were evaluated spectrophotometrically at room temperature using Ellman’s method as described in the method section, with the rate of increase in absorbance at 410 nm followed for 5 min. The experiments were done in triplicate. The tested concentrations of AChE and BChE ranged from 0–84 µM and 0–42 µM respectively. Lineweaver–Burk plots were used to determine the type of inhibition. It was found that the drug inhibits AChE by non-competitive inhibition (Fig. 6A), while for BChE Ondansetron exhibits mixed inhibition (Fig. 6B). The data thus confirm the analysis from the docking regarding the type of inhibition (Fig. 3). The K_m_ of AChE was constant in the presence and absence of the drug and was calculated to ∼ 0.27 mM, while the K_m_ of BChE was variable depending on concentrations of the drug.

#### Affinity of Ondansetron in the enzymes

**Figure 7:**
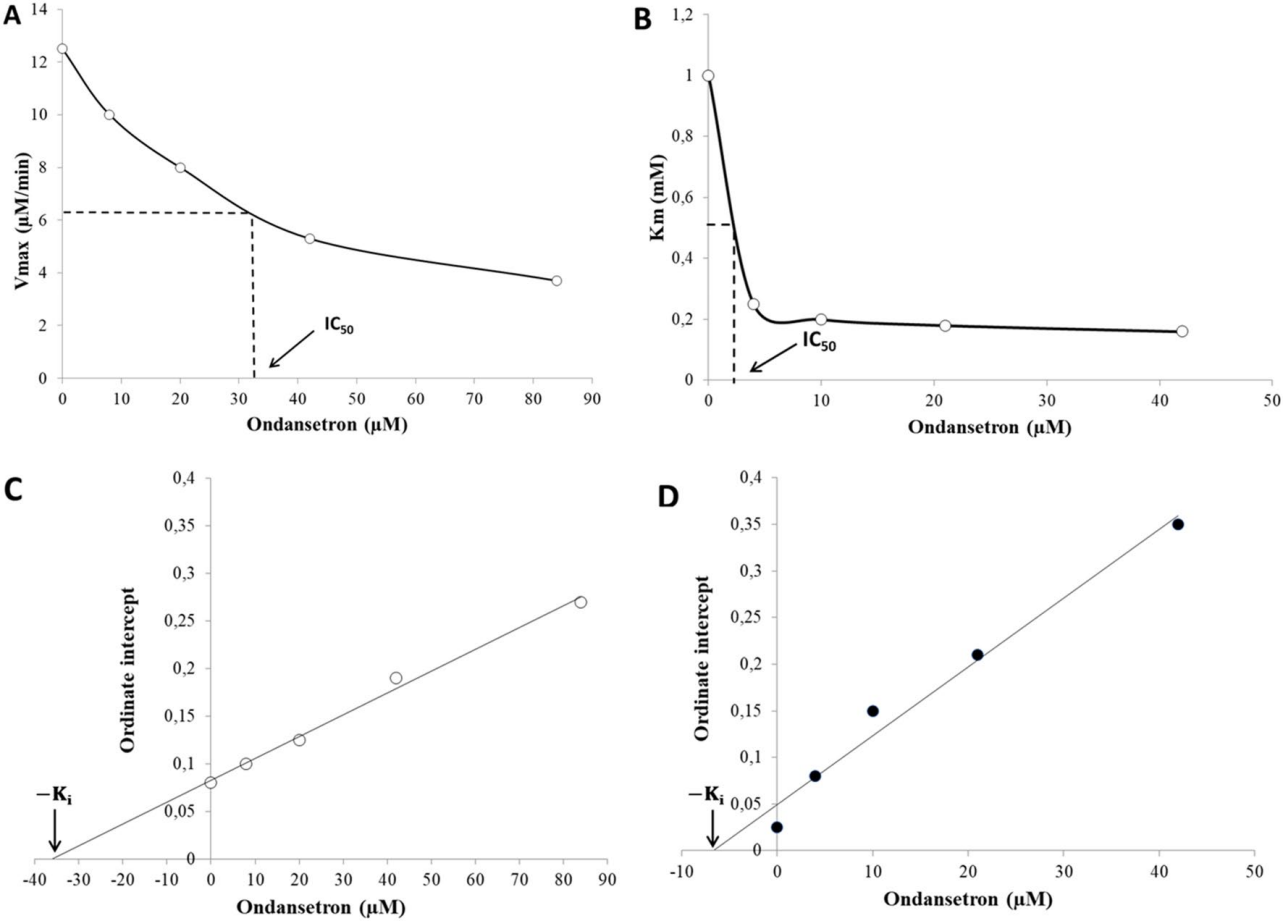
IC_50_ values for Ondansetron in (**A**) AChE, and (**B**) BChE. The secondary plot shows the K_i_ values in (**C**) AChE, and (**D**) BChE.

Since the K_m_ of AChE in the presence of the drug was constant, V_max_ was used to calculate IC_50_ in this case (Fig. 7A). For BChE, K_m_ of mixed inhibition was instead used to determine the IC_50_ value (Fig. 7B). The IC_50_ values thus obtained are 33 µM and 2.5 µM for AChE and BChE, respectively. This can be compared to the IC_50_ values of the reference inhibitors: Rivastigmine was reported to have an IC_50_ value in AChE of 71 μM^45^ or 501 μM^46^, whereas for Tacrine the values range between 0.02 and 0.45 μM^46,47^. Ondansetron is hence a more potent binder to AChE than Rivastigmine, but less so than Tacrine, which agrees perfectly with the MMGBSA data of Table 2. The IC_50_ values for Rivastigmine and Tacrine in BChE were reported to be 7.72–19.95 μM and 0.01-0.03 μM, respectively^46,47,48,49^, indicating that Ondansetron is at least 3–8 times more potent than Rivastigmine but again less effective than Tacrine. Again, this trend is fully supported by the computed binding energies (Table 2). We note however that the most important adverse effect of Tacrine is liver toxicity, gastrointestinal problem, nausea, and vomiting and that it due to this has been withdrawn in some countries^11^. We thus propose that Ondansetron may be a more potent alternative drug targeting cholinesterase than Rivastigmine, and with relatively mild side effects as compared to Tacrine.

Calculation of the inhibition constants (K_i_) were carried out through Lineweaver–Burk and their secondary plots (Fig. 7C,D). The x-intercept indicates the K_i_ value, giving K_i_ = 35 µM and 6.1 µM for Ondansetron in AChE and BChE, respectively, which also confirms the higher binding affinity of Ondansetron in BChE noted computationally.

#### Effect of pH on Ondansetron binding

Both cholinesterases are pH dependent and perform optimally at pH above 7^50^. In general, the rate of enzymatic hydrolysis of acetylcholine and butyrylcholine increases from pH 6 to 9 and shows maximum activity at pH 8. However, the activity decreases markedly at pH 11^51^. Several physiological and pathophysiological conditions contribute to the pH in the neurons. Both an ionic site and positive groups on the surface of ACh receptors recognize this protein for the attachment (Fig. 1) as a result both groups are involved in modulating the pH value in neuron cells. Moreover, some selective ions in the membranes of nerve cells contribute to fixing alkaline charges^52^.

**Figure 8:**
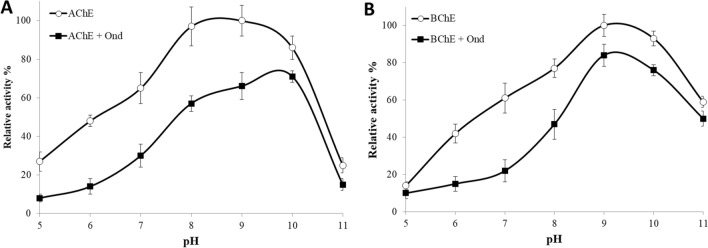
Effect Ondansetron binding at different pH, on the activity of (**A**) AChE and (**B**) BChE. The final concentration of the drug was 21 µM.

To this end, the activity of AChE and BChE was investigated within the pH range 5–11 at 30 °C, using 21 µM Ondansetron. The optimum activity for AChE alone was observed at pH 9, while in the presence of the drug, a shift to pH 10 was observed (Fig. 8A). For BChE the optimum pH is 9, which remained unaltered in the presence of the drug (Fig. 8B). The pH profiles hence suggest that both enzymes are more active in alkaline pH than in acidic pH, and also show a clear impact of drug binding on the enzymatic activity.

#### Effect of temperature on Ondansetron binding

**Figure 9:**
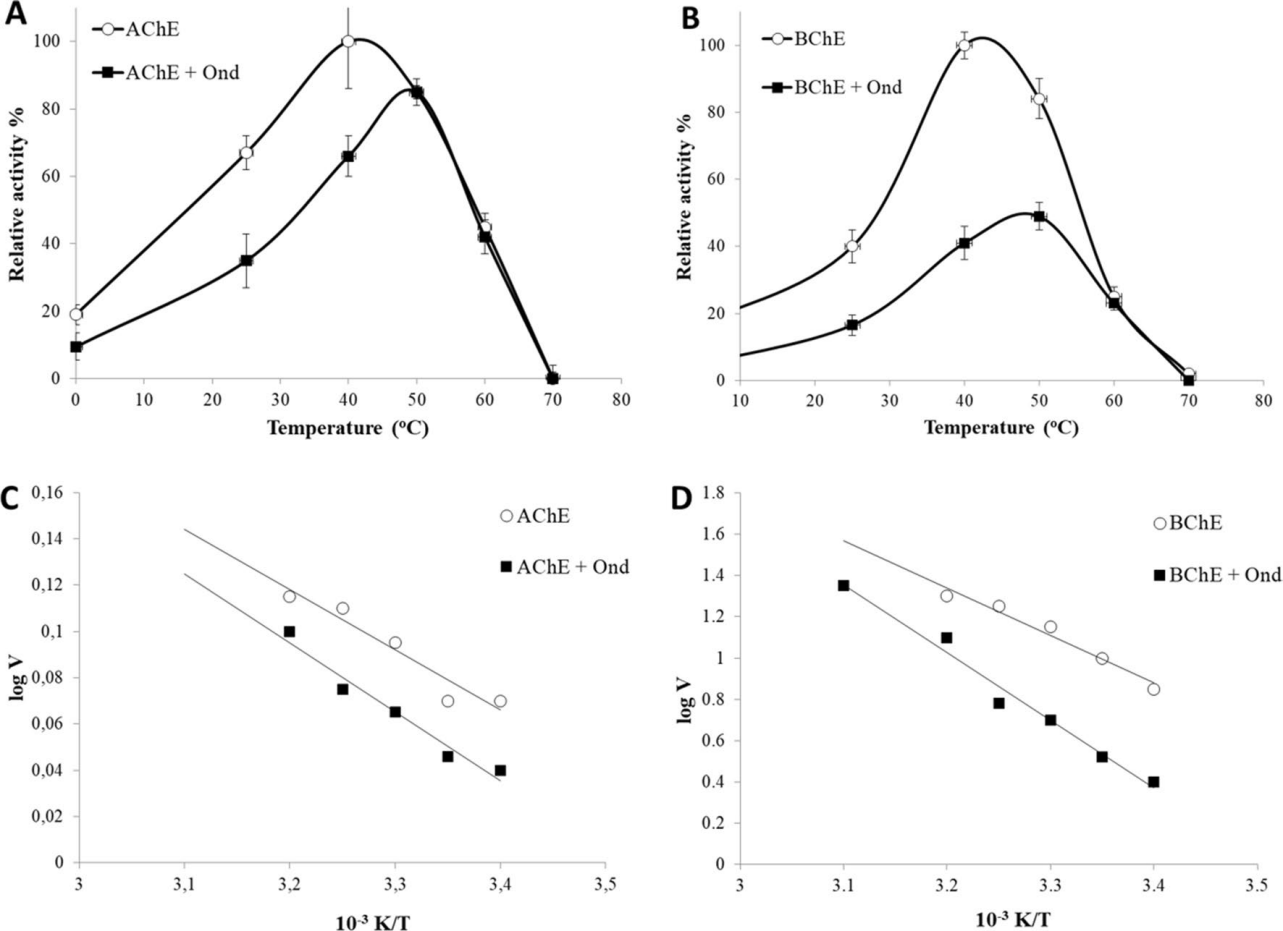
Effect of different temperatures on the activity of (**A**) AChE, and (**B**) BChE in absence and presence of 21 µM Ondansetron. Arrhenius plot for (**C**) AChE, and (**D**) BChE with and without presence of the drug.

The effect of different temperatures (0–70 °C) on the enzymatic activity was also measured in the presence (21 µM concentration) and absence of Ondansetron. AChE showed maximum activity at 40 °C and 50 °C in the absence and presence of the drug, respectively (Fig. 9A). BChE showed the same pattern as AChE, with optimum activity at 40 °C while in the presence of Ondansetron it shifted to 50 °C (Fig. 9B). Both enzymes were also inactivated at 70 °C both in presence and absence of the drug.

Arrhenius plots were obtained to calculate the activation energies of the enzymes and the effect of the drugs thereon. It indicated that, in the presence of Ondansetron, more energy was needed for the reaction to proceed, suggesting that binding of the drug to the enzymes will increase the activation energy. As a result, Vmax of the enzymes were calculated at different temperatures in order to generate Arrhenius plots. For AChE, Vmax increased from 25 to 30 kJ mol^−1^ in the presence of the drug (Fig. 9C) while for BChE the activation energy was 23 and 32 kJ mol^−1^ in the absence and presence of the drug respectively (Fig. 9D). Again, this supports the finding of Ondansetron binding stronger to BChE, as noted in Table 2.

## Conclusions

Ondansetron has herein for the first time been investigated as a potent inhibitor for cholinesterase proteins. In the current study, computational modelling and experimental studies were carried out to explore structural properties and binding mechanisms of the compound. Our observations suggest that it can inhibit the enzyme activity by interaction with residues near or in the active site of the enzymes, in non-competitive or mixed inhibition manners for AChE and BChE, respectively. Analyses of MD simulations revealed that Ondansetron is a stable ligand in both enzymes, and binds with higher affinity in comparison with Rivastigmine. We also carried out different experimental studies by calculating the type of inhibition and K_i_ values from Lineweaver–Burk plots that fully confirm the data from the in silico modeling. The effect of binding to AChE and BChE were investigated, that show that our target has stronger inhibitory activity towards BChE than AChE (IC_50_: 2.5 µM and 33 µM, respectively). However, in relation to the two FDA approved drugs Rivastigmine and Tacrine, and considering the serious side effects such as hepatotoxicity for Tacrine, we herein propose that Ondansetron may be a potential repurposed candidate drug to reduce or block cholinesterase activity. Further experimental studies (in vitro and in vivo) would be required to verify the pharmacological relevance of the observed cholinesterase inhibition by Ondansetron.

## Supplementary Information

Supplementary Figures.

## Data Availability

All docked structures, MD simulation trajectories and BPMD data are available as free download at Zenodo.org, 10.5281/zenodo.7003730.

## References

[1] Sahin K, Zengin Kurt B, Sonmez F, Durdagi S (2020). Novel AChE and BChE inhibitors using combined virtual screening, text mining and in vitro binding assays. J. Biomol. Struct. Dyn..

[2] Anand P, Singh B (2013). A review on cholinesterase inhibitors for Alzheimer’s disease. Arch. Pharm. Res..

[3] Košak U (2016). Development of an in-vivo active reversible butyrylcholinesterase inhibitor. Sci. Rep..

[4] Vecchio I, Sorrentino L, Paoletti A, Marra R, Arbitrio M (2021). The state of the art on acetylcholinesterase inhibitors in the treatment of Alzheimer’s disease. J. Cent. Nerv. Syst. Dis..

[5] Zhou Y (2019). Discovery of selective butyrylcholinesterase (BChE) inhibitors through a combination of computational studies and biological evaluations. Molecules.

[6] Cavdar H (2019). Inhibition of acetylcholinesterase and butyrylcholinesterase with uracil derivatives: Kinetic and computational studies. J. Enzyme Inhib. Med. Chem..

[7] Sarkar B, Alam S, Rajib TK, Islam SS, Araf Y, Ullah M (2021). Identification of the most potent acetylcholinesterase inhibitors from plants for possible treatment of Alzheimer’s disease: A computational approach. Egypt. J. Med. Hum. Genet..

[8] Waymire, J. C. Chapter 11: Acetylcholine neurotransmission. *Neurosci. Online* (2000).

[9] Andrisano V, Naldi M, De Simone A, Bartolini M (2018). A patent review of butyrylcholinesterase inhibitors and reactivators 2010–2017. Expert Opin. Ther. Pat..

[10] Seniya, C., Khan, G. J. & Uchadia, K. Identification of potential herbal inhibitor of acetylcholinesterase associated Alzheimer’s disorders using molecular docking and molecular dynamics simulation. *Biochem. Res. Int.* **2014** (2014). 10.1155/2014/705451 PMC4099354 25054066

[11] Pohanka M (2014). Inhibitors of acetylcholinesterase and butyrylcholinesterase meet immunity. Int. J. Mol. Sci..

[12] Haake A, Nguyen K, Friedman L, Chakkamparambil B, Grossberg GT (2020). An update on the utility and safety of cholinesterase inhibitors for the treatment of Alzheimer’s disease. Expert Opin. Drug Saf..

[13] Marucci G, Buccioni M, Dal Ben D, Lambertucci C, Volpini R, Amenta F (2021). Efficacy of acetylcholinesterase inhibitors in Alzheimer’s disease. Neuropharmacology.

[14] Allahham N (2020). Selective laser sintering 3D printing of orally disintegrating printlets containing ondansetron. Pharmaceutics.

[15] Sandhya B, Hegde AH, Ramesh KC, Seetharamappa J (2012). Exploring the binding mechanism of ondansetron hydrochloride to serum albumins: Spectroscopic approach. Spectrochim. Acta Part A Mol. Biomol. Spectrosc..

[16] Parker SE, Van Bennekom C, Anderka M, Mitchell AA (2018). Ondansetron for treatment of nausea and vomiting of pregnancy and the risk of specific birth defects. Obstet. Gynecol..

[17] Ye J, Ponnudurai R, Schaefer R (2001). Ondansetron: A selective 5-HT3 receptor antagonist and its applications in CNS-related disorders. CNS Drug Rev..

[18] Capacio BR, Byers CE, Anderson DR, Matthews RL, Brown DE (1996). The effect of ondansetron on pyridostigmine-induced blood acetylcholinesterase inhibition in the guinea pig. Drug Chem. Toxicol..

[19] Skovgård K, Agerskov C, Kohlmeier KA, Herrik KF (2018). The 5-HT3 receptor antagonist ondansetron potentiates the effects of the acetylcholinesterase inhibitor donepezil on neuronal network oscillations in the rat dorsal hippocampus. Neuropharmacology.

[20] Stern ER (2019). High-dose ondansetron reduces activation of interoceptive and sensorimotor brain regions. Neuropsychopharmacology.

[21] Kappenberg YG (2022). Design, synthesis, AChE/BChE inhibitory activity, and molecular docking of spiro [chromeno[4,3-b]thieno[3,2-e]pyridine]-7-amine tacrine hybrids. J. Mol. Struct..

[22] Cheung J, Gary EN, Shiomi K, Rosenberry TL (2013). Structures of human acetylcholinesterase bound to dihydrotanshinone I and territrem B show peripheral site flexibility. ACS Med. Chem. Lett..

[23] Brazzolotto X (2012). Human butyrylcholinesterase produced in insect cells: Huprine-based affinity purification and crystal structure. FEBS J..

[24] Madhavi Sastry G, Adzhigirey M, Day T, Annabhimoju R, Sherman W (2013). Protein and ligand preparation: Parameters, protocols, and influence on virtual screening enrichments. J. Comput. Aided. Mol. Des..

[25] Roos K (2019). OPLS3e: Extending force field coverage for drug-like small molecules. J. Chem. Theory Comput..

[26] Greenwood JR, Calkins D, Sullivan AP, Shelley JC (2010). Towards the comprehensive, rapid, and accurate prediction of the favorable tautomeric states of drug-like molecules in aqueous solution. J. Comput. Aided. Mol. Des..

[27] Sherman W, Day T, Jacobson MP, Friesner RA, Farid R (2006). Novel procedure for modeling ligand/receptor induced fit effects. J. Med. Chem..

[28] Farid R, Day T, Friesner RA, Pearlstein RA (2006). New insights about HERG blockade obtained from protein modeling, potential energy mapping, and docking studies. Bioorg. Med. Chem..

[29] Filimonov DA (2014). Prediction of the biological activity spectra of organic compounds using the PASS online web resource. Chem. Heterocycl. Compd..

[30] Bowers, K. J. *et al.* Scalable algorithms for molecular dynamics simulations on commodity clusters. In *SC’06: Proceedings of the 2006 ACM/IEEE Conference on Supercomputing*, 43 (2006).

[31] Jorgensen WL, Chandrasekhar J, Madura JD, Impey RW, Klein ML (1983). Comparison of simple potential functions for simulating liquid water. J. Chem. Phys..

[32] Martyna GJ, Klein ML, Tuckerman M (1992). Nosé–Hoover chains: The canonical ensemble via continuous dynamics. J. Chem. Phys..

[33] Wentzcovitch RM (1991). Invariant molecular-dynamics approach to structural phase transitions. Phys. Rev. B.

[34] Fusani L, Palmer DS, Somers DO, Wall ID (2020). Exploring ligand stability in protein crystal structures using binding pose metadynamics. J. Chem. Inf. Model..

[35] Ellman GL, Courtney KD, Andres V, Featherstone RM (1961). A new and rapid colorimetric determination of acetylcholinesterase activity. Biochem. Pharmacol..

[36] Jahangirvand M, Minai-Tehrani D, Yazdi F, Minai-Tehrani A, Razmi N (2016). Binding of cimetidine to Balb/C mouse liver catalase; kinetics and conformational studies. Curr. Clin. Pharmacol..

[37] Yazdi F (2015). Functional and structural changes of human erythrocyte catalase induced by cimetidine: Proposed model of binding. Mol. Cell. Biochem..

[38] Lowry O, Rosebrough N, Farr AL, Randall R (1951). Protein measurement with the Folin phenol reagent. J. Biol. Chem..

[39] Bajda M, Więckowska A, Hebda M, Guzior N, Sotriffer CA, Malawska B (2013). Structure-based search for new inhibitors of cholinesterases. Int. J. Mol. Sci..

[40] Gao X, Zhou C, Liu H, Liu L, Tang J, Xia X (2017). Tertiary amine derivatives of chlorochalcone as acetylcholinesterase (AChE) and buthylcholinesterase (BuChE) inhibitors: The influence of chlorine, alkyl amine side chain and α, β-unsaturated ketone group. J. Enzyme Inhib. Med. Chem..

[41] Rosenberry TL (2017). Comparison of the binding of reversible inhibitors to human butyrylcholinesterase and acetylcholinesterase: A crystallographic, kinetic and calorimetric study. Molecules.

[42] Darvesh S (2008). Carbamates with differential mechanism of inhibition toward acetylcholinesterase and butyrylcholinesterase. J. Med. Chem..

[43] Lagunin A, Stepanchikova A, Filimonov D, Poroikov V (2000). PASS: Prediction of activity spectra for biologically active substances. Bioinformatics.

[44] Geronikaki A, Poroikov V, Hadjipavlou-Litina D, Filimonov D, Lagunin A, Mgonzo R (1999). Computer aided predicting the biological activity spectra and experimental testing of new thiazole derivatives. Quant. Struct. Relationships.

[45] David B, Schneider P, Schäfer P, Pietruszka J, Gohlke H (2021). Discovery of new acetylcholinesterase inhibitors for Alzheimer’s disease: Virtual screening and in vitro characterisation. J. Enzyme Inhib. Med. Chem..

[46] Krátký M, Štěpánková Š, Vorčáková K, Švarcová M, Vinšová J (2016). Novel cholinesterase inhibitors based on O-aromatic N, N-disubstituted carbamates and thiocarbamates. Molecules.

[47] Zawada K (2021). New hybrids of tacrine and indomethacin as multifunctional acetylcholinesterase inhibitors. Chem. Pap..

[48] SadafiKohnehshahri M (2022). Novel tacrine-based acetylcholinesterase inhibitors as potential agents for the treatment of Alzheimer’s disease: Quinolotacrine hybrids. Mol. Divers..

[49] Scheiner M (2019). Dual-acting cholinesterase–human cannabinoid receptor 2 ligands show pronounced neuroprotection in vitro and overadditive and disease-modifying neuroprotective effects in vivo. J. Med. Chem..

[50] Wessler I, Michel-Schmidt R, Kirkpatrick CJ (2015). pH-dependent hydrolysis of acetylcholine: Consequences for non-neuronal acetylcholine. Int. Immunopharmacol..

[51] Eränkö L (1972). Effect of pH on the activity of nervous cholinesterases of the rat towards different biochemical and histochemical substrates and inhibitors. Histochemie.

[52] Dunning BB, Machne X (1971). pH optimum for the rate of acetylcholine action on neurons. Agents Actions.

