# Supplementary material for: In silico and in vitro studies confirm Ondansetron as a novel acetylcholinesterase and butyrylcholinesterase inhibitor
Source: Sci Rep. 2023 Jan 12;13:643. doi: 10.1038/s41598-022-27149-z (PMC9837033; doi:10.1038/s41598-022-27149-z)
Supplement: Supplementary file 1 — Supplementary Figures. [file 41598_2022_27149_MOESM1_ESM.pdf]

In silico and *in vitro* Studies Confirm Ondansetron as a Novel Acetylcholinesterase and Butyrylcholinesterase Inhibitor

Asma Gholami[1,2], *Dariush Minai-Tehrani*[1*], *Leif A. Eriksson*[2*]

[1]Faculty of Life Sciences and Biotechnology, Shahid Beheshti University, Tehran, Iran

[2]Department of chemistry and molecular biology, University of Gothenburg, 405 30 Göteborg, Sweden

*Corresponding authors: leif.eriksson@chem.gu.se, d_mtehrani@sbu.ac.ir,

SUPPLEMENTARY MATERIAL

Figure S1: Sequence alignment of AChE and BChE.

Figure S2: Superposition of the co-crystallized ligands of AChE and BChE with the corresponding docked poses.

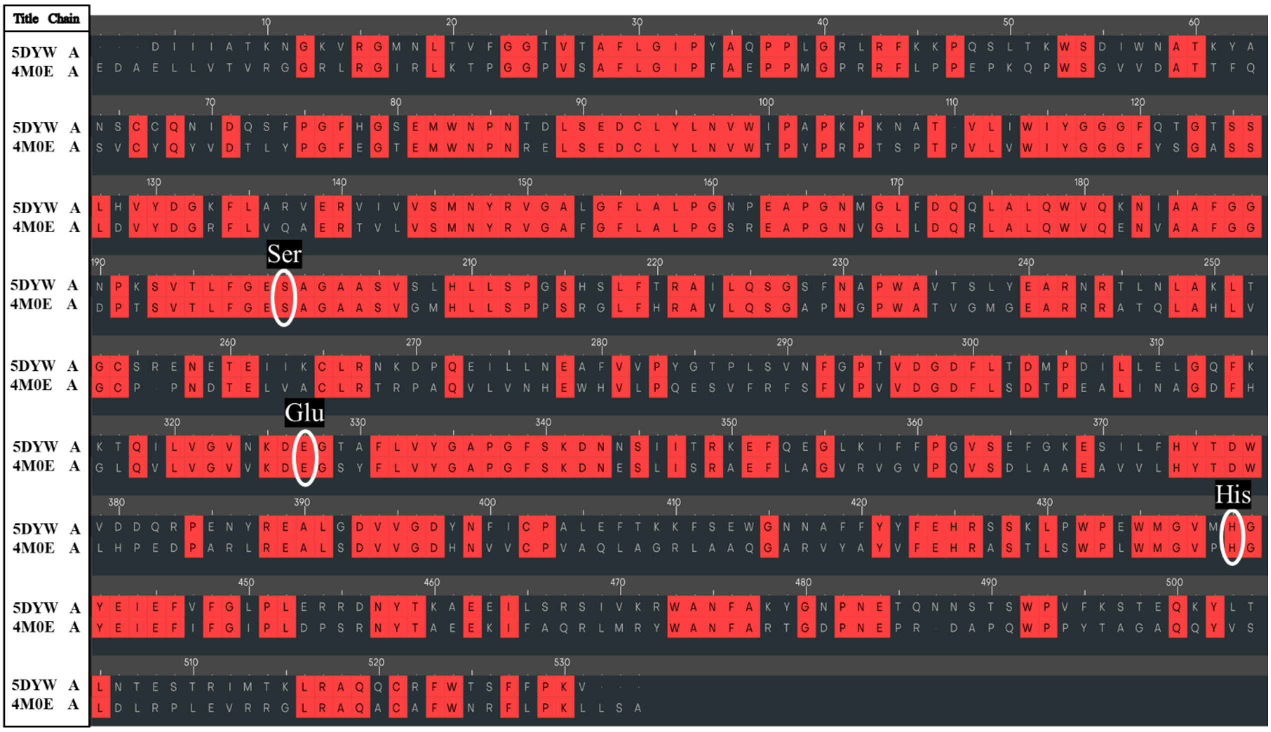

Figure S1. Alignment of AChE (PDB ID 4M0E) and BChE (PDB ID 5DYW) sequences from Homo sapiens. White circles are conserved residues in the active sites of the two enzymes. Red color indicates identical residues.

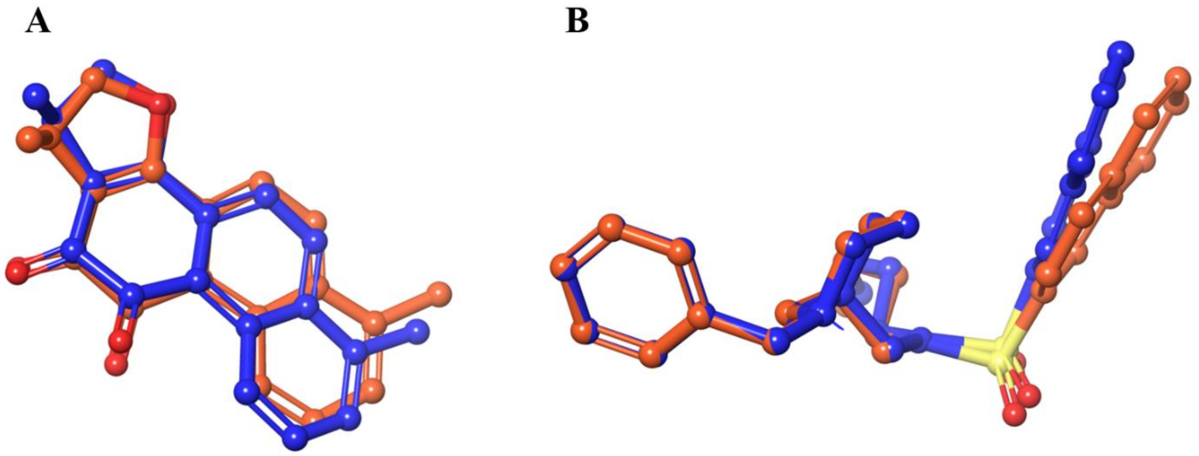

Figure S2. Superposition of the docked pose (orange) of the bound ligand with its co-crystallized conformation (blue) in the crystal structure of (A) AChE (PDB ID:4M0E), and (B) BChE (PDB ID: 5DWY).
